# Supplementary material for: Sertm2 is a conserved micropeptide that promotes GDNF-mediated motor neuron subtype specification
Source: EMBO Rep. 2025 Mar 19;26(8):2013–43. doi: 10.1038/s44319-025-00400-0 (PMC12018958; doi:10.1038/s44319-025-00400-0)
Supplement: Supplementary file 1 — Table EV1 [file 44319_2025_400_MOESM1_ESM.docx]

**Table EV1. Oligonucleotides and sequence-based reagents**

| Oligonucleotides | Sequence |
| --- | --- |
| Primer for genotyping | |
| Mycoplasma-F | GGGAGCAAACAGGATTAGATACCCT |
| Mycoplasma-R | TGCACCATCTGTCACTCTGTTAACCTC |
| Mouse: Sertm2 N-F | ACACATGCATTCGCTGTTTGC |
| Mouse: Sertm2 C-F | GTTCTGTGGCAAAACTGGAAC |
| Mouse: Sertm2 C-R | TGTGTGCGTGATCTCACTTG |
| Mouse: Sertm2 KI-F | TGATGACGGAGGTACTTTTTAAGTATCATGG |
| Mouse: Sertm2 KI-R | CTCTTGAGCCTCTTAGAGGTCCC |
| Human: SERTM2 WT-F1 | CGCAGAAGAGCCCAATTAAG |
| Human: SERTM2 WT-R1 | GCTTTAGGGAGTGGGGATTC |
| Human: SERTM2 WT-F2 | ATCCATGCCAAATCTGAAGC |
| Human: SERTM2 WT-R2 or  KO-R | GGAATGTGGAAGGCTATTGG |
| Human: SERTM2 KO-F | TCACATCCTGGAATGTTTGG |
|  |  |
| Primer for gene editing | |
| Mouse: Sertm2 KO sgRNA-N | CCCATTTTGCCGTGAGAGCT NGG |
| Mouse: Sertm2 KO sgRNA-C | TTGGAGGCCTCATTAGTATT NGG |
| Mouse: Sertm2 KI sgRNA | ATAATCCTGACTTTAAGGAG NGG |
| Mouse: Sertm2-Flag KI ssODN | AGAGATGCAAAGTCGAATTCCCACTCCTGACTACAAAGACCATGACGGTGATTATAAAGATCATGACATCGATTACAAGGATGACGATGACAAGTAAAGTCAGGATTATGCTGAGACTTGGCGGATAGA |
| Human: SERTM2 KO sgRNA-N | CACCGTTCATGAGGAAGCCTCTAAG NGG |
| Human: SERTM2 KO sgRNA-C | CACCGTGCACCAATCACTTGACAAG NGG |
|  |  |
| Primers for construct | |
| ATG-Sertm2(ORF)-F | GACGCGGCCGCATGACGGAGGTACTTTTTA |
| mut-ATG-Sertm2(ORF)-F | GACGCGGCCGCATTACGGAGGTACTTTTTA |
| Sertm2(ORF)-Flag-STOP-R | AATTGGATCCTTACTTGTCGTCATCGTCTTTGTAGTCTCCTCCAGGAGTGGGAATT |
| Primer for *in situ* hybridization probe | |
| A730046J19Rik/Sertm2-ISH-F | ATGCTGAGCCTGAGATACCC |
| A730046J19Rik/Sertm2-ISH-R | GAGTAATACGACTCACTATAGGGCAGGGTTTGCTTCTATCCGC |
|  |  |
| Primer for qPCR | |
| Mouse: Sertm2 (UTR)-F | CCAGCCGTACTCATTCTGTG |
| Mouse: Sertm2 (UTR)-R | GTAGGCTGAAGGGAAGGTGT |
| Mouse: Sertm2 (ORF)-F | CAGAGGCTGACACCACTTCA |
| Mouse: Sertm2 (ORF)-R | GGAGTGGGAATTCGACTTTG |
| Mouse: Sertm2 (ORF-KI)-F | CACAATGCTTCTTCGGCTCA |
| Mouse: Sertm2 (ORF-KI)-R | ACTTGTCATCGTCATCCTTGT |
| Mouse: Etv4-F | GGTACCGGACAGTGATGAGC |
| Mouse: Etv4-R | AGGCACTGGAGTAAAGGCAC |
| Mouse: Mnx1-F | GTTGGAGCTGGAACACCAGT |
| Mouse: Mnx1-R | GCTCTTTGGCCTTTTTGCT |
| Mouse: Hoxc8-F | GTAAATCCTCCGCCAACACTAA |
| Mouse: Hoxc8-R | CGCTTTCTGGTCAAATAAGGAT |
| Mouse: Dcx-F | GAAAACAGCCCACTCTTTCG |
| Mouse: Dcx-R | GCATAGCGGAATTTTTCAGG |
| Mouse: Alg13-F | GCAGGAAGCTGTTTGGAGAG |
| Mouse: Alg13-R | ACTGTAACAGCCCAGGAAGC |
| Mouse: Gapdh-F | TGACCACAGTCCATGCCATC |
| Mouse: Gapdh-R | GACGGACACATTGGGGGTAG |
| Human: GAPDH-F | TGACCACAGTCCATGCCATC |
| Human: GAPDH-R | GACGGACACATTGGGGGTAG |
| Human: U1-F | TGGCAGGGCAGATACCAT |
| Human: U1-R | GCCATCGCTCACTACCAAAA |
| Human: SERTM2-F | ATTCATACTGTGGGCCAGGA |
| Human: SERTM2-R | TGTAGGCTGGAGGGAAAGTG |
| Human: ETV4-F | GGCAATTTCTGGTGGCCTTG |
| Human: ETV4-R | CGACCTCCTCAGGCTCAATG |
| Human: FOXP1-F | CAAACAACCAGCTCTTCAGGT |
| Human: FOXP1-R | CTGAGGGCTCAGCACTTGT |
