# Supplementary material for: Sertm2 is a conserved micropeptide that promotes GDNF-mediated motor neuron subtype specification
Source: EMBO Rep. 2025 Mar 19;26(8):2013–43. doi: 10.1038/s44319-025-00400-0 (PMC12018958; doi:10.1038/s44319-025-00400-0)
Supplement: Supplementary file 4 — Movie EV2 [file 44319_2025_400_MOESM4_ESM.zip › EMBOR-2024-60252_Movie EV2/Movie EV2.rtf]

Movie EV2: (Related Figures 5 and EV4) Ctrl and Sertm2 KO mice performed a treadmill test at P30.
