# Supplementary material for: Sertm2 is a conserved micropeptide that promotes GDNF-mediated motor neuron subtype specification
Source: EMBO Rep. 2025 Mar 19;26(8):2013–43. doi: 10.1038/s44319-025-00400-0 (PMC12018958; doi:10.1038/s44319-025-00400-0)
Supplement: Supplementary file 5 — Expanded View Figures [file 44319_2025_400_MOESM5_ESM.pdf]

Expanded View Figures

Figure EV1. Features and expression of *A730046J19Rik* in the MMC and LMC of the brachial spinal cord, along with protein-coding potential and peptide validation of human SERTM2.

(Related to Fig. 1). (**A**) Multiple sequence alignments of *A730046J19Rik* reveal high conservation across four vertebrate species. The 20-way El track represents conserved elements among 17 primates and three other mammals. The conservation tracks were sourced from the UCSC Genome Browser. (**B**) UMAP visualization displaying *A730046J19Rik, Nr2f2, Satb2*, and *Bcl11b* expression in MMC MN subtypes. The dataset is derived from Liau et al, 2023. (**C**) RNAscope-based ISH of *A730046J19Rik, Satb2, and Nr2f2* in the E13.5 B6 brachial spinal cord. Dashed lines outline the spinal cord boundary. Scale bar, 20 µm. (**D**) Scatter plots depicting correlations of gene expression in brachial LMC neurons, with the Pearson correlation coefficient indicated above each plot. (**E**) Mass spectrometry identified one unique peptide specific to human SERTM2 in human tissue. The data was downloaded from Liu et al, 2022 (LncPep). (**F**) Protein-coding potential of human *SERTM2*, as predicted by the PhyloCSF database.

 © The Author(s)

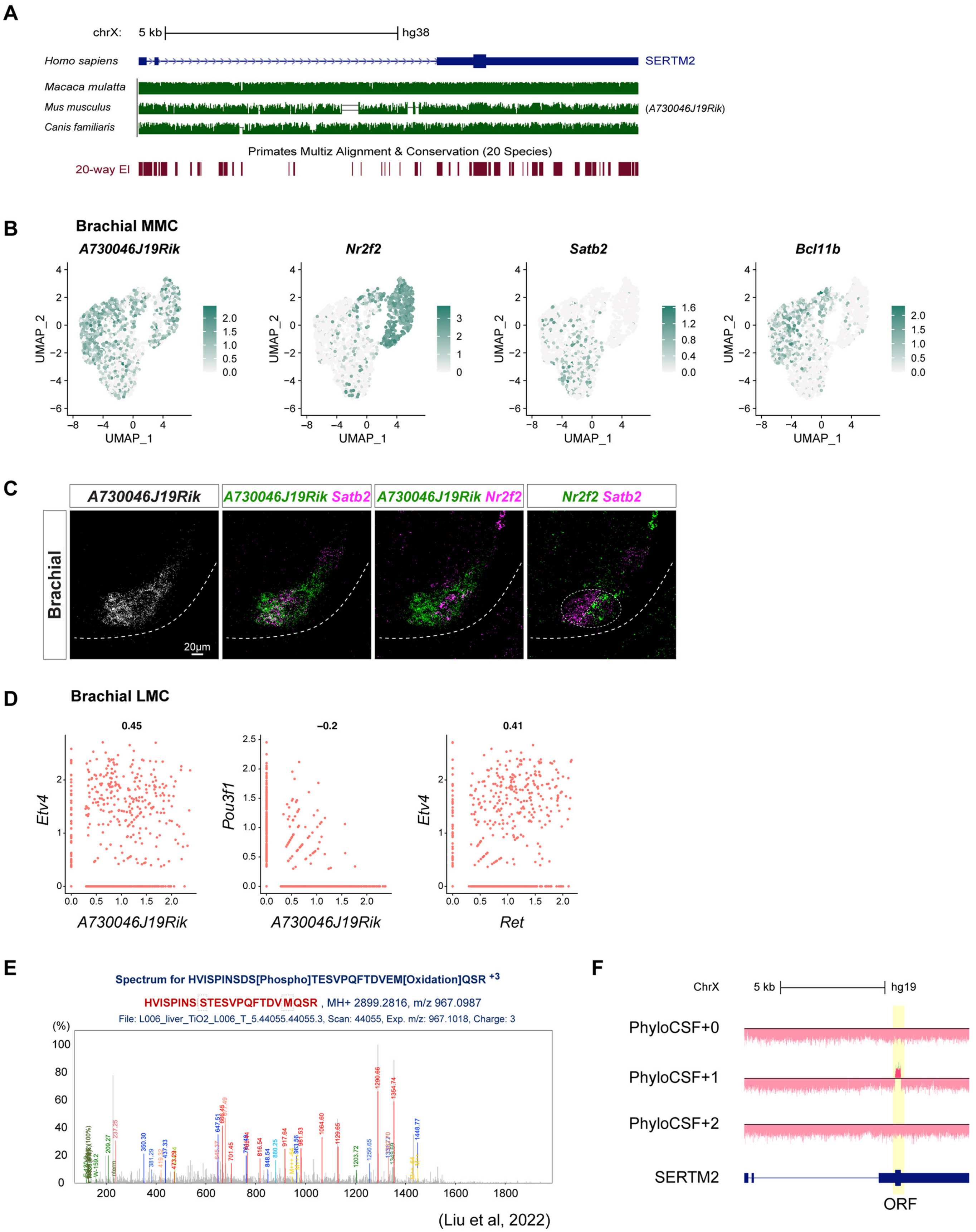
A
chrX: 5 kb hg38
Homo sapiens
SERTM2
Macaca mulatta
Mus musculus
(A730046J19Rik)
Canis familiaris
Primates Multiz Alignment & Conservation (20 Species)
20-way El
B
Brachial MMC
A730046J19Rik
Nr2f2
Satb2
Bcl11b
UMAP_1
UMAP_2
C
A730046J19Rik
A730046J19Rik Satb2
A730046J19Rik Nr2f2
Nr2f2 Satb2
Brachial
20μm
D
Brachial LMC
0.45
−0.2
0.41
Etv4
Pou3f1
A730046J19Rik
Ret
E
Spectrum for HVISPINSDS[Phospho]TESVPQFTDVEM[Oxidation]QSR +3
HVISPINS STESVPQFTDV MQSR , MH+ 2899.2816, m/z 967.0987
File: L006_liver_TiO2_L006_T_5.44055.44055.3, Scan: 44055, Exp. m/z: 967.1018, Charge: 3
(%)
(Liu et al, 2022)
F
ChrX 5 kb hg19
PhyloCSF+0
PhyloCSF+1
PhyloCSF+2
SERTM2
ORF

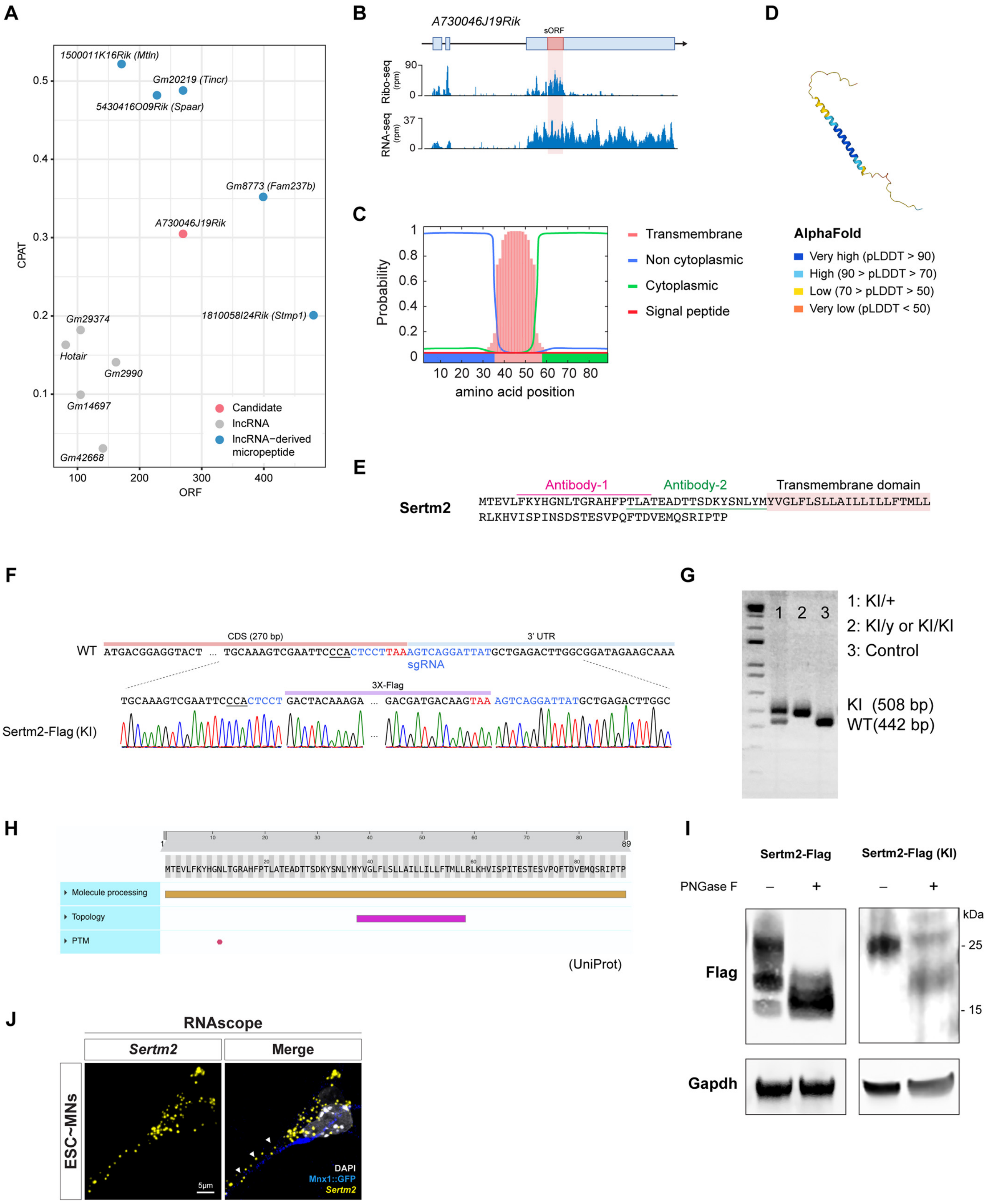
A
1500011K16Rik (Mtln)
Gm20219 (Tincr)
5430416O09Rik (Spaar)
Gm8773 (Fam237b)
A730046J19Rik
Gm29374
1810058I24Rik (Stmp1)
Hotair
Gm2990
Gm14697
Gm42668
CPAT
ORF
Candidate
lncRNA
lncRNA–derived micropeptide
B
A730046J19Rik
sORF
Ribo-seq (rpm)
RNA-seq (rpm)
C
Probability
amino acid position
Transmembrane
Non cytoplasmic
Cytoplasmic
Signal peptide
D
AlphaFold
Very high (pLDDT > 90)
High (90 > pLDDT > 70)
Low (70 > pLDDT > 50)
Very low (pLDDT < 50)
E
Antibody-1
Antibody-2
Transmembrane domain
Sertm2
MTEVLFKYHGNLTGRAHFPTLATEADTTSDKYSNLYMYVGLFLSLLAILLILLFTMLL
RLKHVISPINSDSTESVPQFTDVEMQSRIPTP
F
CDS (270 bp)
3' UTR
WT ATGACGGAGGTACT ... TGCAAAGTCGAATTCCCACTCCTTAAAGTCAGGATTATGCTGAGACTTGGCGGATAGAAGCAAA
sgRNA
3X-Flag
TGCAAAGTCGAATTCCCACTCCT GACTACAAAGA ... GACGATGACAAGTAA AGTCAGGATTATGCTGAGACTTGGC
Sertm2-Flag (KI)
G
1 2 3
1: KI/+
2: KI/y or KI/KI
3: Control
KI (508 bp)
WT(442 bp)
H
MTEVLFKYHGNLTGRAHFPTLATEADTTSDKYSNLYMYVGLFLSLLAILLILLFTMLLRLKHVISPITESTESVPQFTDVEMQSRIPTP
Molecule processing
Topology
PTM
(UniProt)
I
Sertm2-Flag
Sertm2-Flag (KI)
PNGase F
kDa
- 25
- 15
Flag
Gapdh
J
RNAscope
Sertm2
Merge
ESC~MNs
5μm
DAPI
Mnx1::GFP
Sertm2

 © The Author(s)

Figure EV2. Investigation of coding potential and functional analysis of mouse *Sertm2*.

(Related to Figs. 2 and 3). (**A**) Protein-coding potential of the indicated lncRNAs, as determined in CPAT (Wang et al, 2013). Scatter plot showing the evidence-supported lncRNA-derived micropeptides (blue), lncRNAs with low coding potential (gray), and *A730046J19Rik* (red). (**B**) Ribosome footprint and mRNA fragment densities for *A730046J19Rik* transcripts, as provided by the GWIPS-viz browser. (**C** and **D**) Protein distribution and structure, as predicted by Phobius (**C**) and AlphaFold (**D**), respectively. (**E**) Design of two epitopes for Sertm2-targeting antibodies. (**F**) Validation of CRISPR/Cas9-mediated Sertm2-Flag KI mice by Sanger sequencing. (**G**) Genotyping of Sertm2-Flag KI mice. (**H**) The UniProt database identifies a glycosylation site on the N-terminal region of Sertm2, represented by a pink hexagon. The pink rectangle indicates the transmembrane domain within Sertm2. (**I**) Sertm2 undergoes extensive N-glycosylation, as demonstrated by PNGase F treatment, which shifts its SDS-PAGE migration from 15–25 kDa to approximately 15 kDa. Sertm2-Flag from in vitro overexpression in HEK293T cells (left) and Sertm2-Flag KI from endogenous expression in the spinal cord (right), with both representing evidence of post-translational modifications (PTMs) of the Sertm2 protein. Flag indicates Sertm2 protein, and Gapdh serves as a loading control. (**J**) RNAscope-based ISH reveals the distribution of *Sertm2* in the axon during ESC~MNs. Motor axons are identified by endogenous Mnx1::GFP, while nuclei are highlighted by DAPI staining. White arrows show the localization of *Sertm2* within motor axons. Scale bar: 5 µm.

© The Author(s)

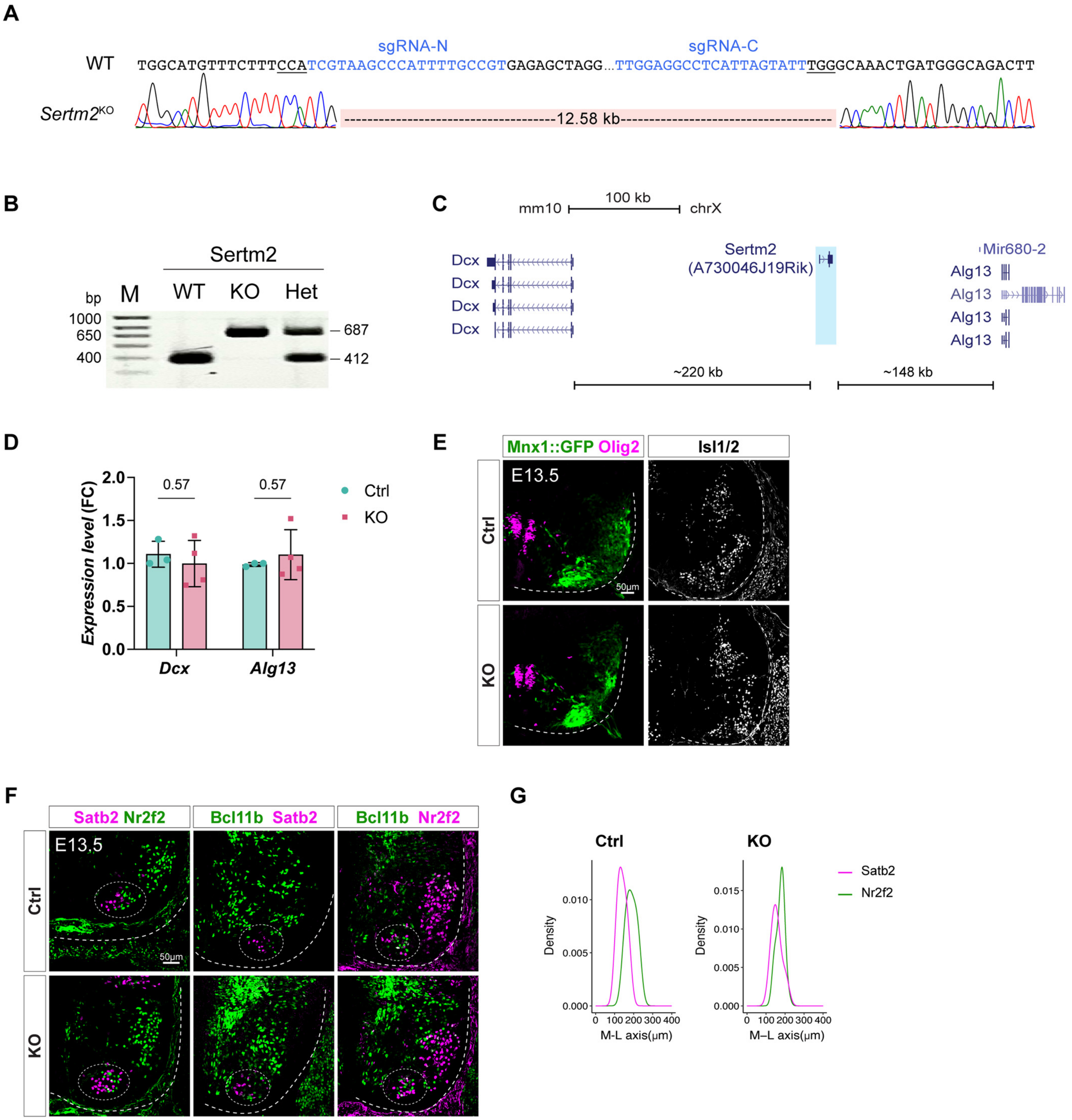

Figure EV3. Analysis of *Sertm2* depletion effects on neighboring gene expression and the MMC population in the spinal cord.

(Related to Fig. 4). (**A**) Accurate CRISPR/Cas9 genome editing of the *Sertm2* locus in ESCs, as confirmed by Sanger sequencing. The design of sgRNAs is highlighted in blue. (**B**) Genotyping of the *Sertm2* knockout cell and mouse lines. (**C**) Graphical representation of the mouse *Sertm2* locus and its neighboring genes, *Dcx* and *Alg13* (mm10). (**D**) Depletion of *Sertm2* does not influence gene expression in *cis*. Data from $n = 3$–4 independent experiments; unpaired two-tailed *t* test. *P* values for *Dcx* and *Alg13* (Ctrl vs. KO) were 0.57 and 0.57. (**E**) Immunodetection of Olig2, Mnx1::GFP, and Isl1/2 in E13.5 brachial spinal cord of Ctrl and KO mice. Dashed lines outline the spinal cord. Scale bar: 50 µm. (**F**) Immunostaining of the MMC subtype markers Bcl11b, Satb2, and Nr2f2 in E13.5 brachial spinal cord. Dashed lines outline the spinal cord boundary, and dashed circles demarcate MMC MNs. Scale bar: 50 µm. (**G**) Mediolateral (M–L) density plot of the Satb2$^+$ and Nr2f2$^+$ subtypes in the E13.5 brachial spinal cord of Ctrl and *Sertm2* mutant mice (KO) in (**F**). Data from $n = 4$ independent biological samples.

 © The Author(s)

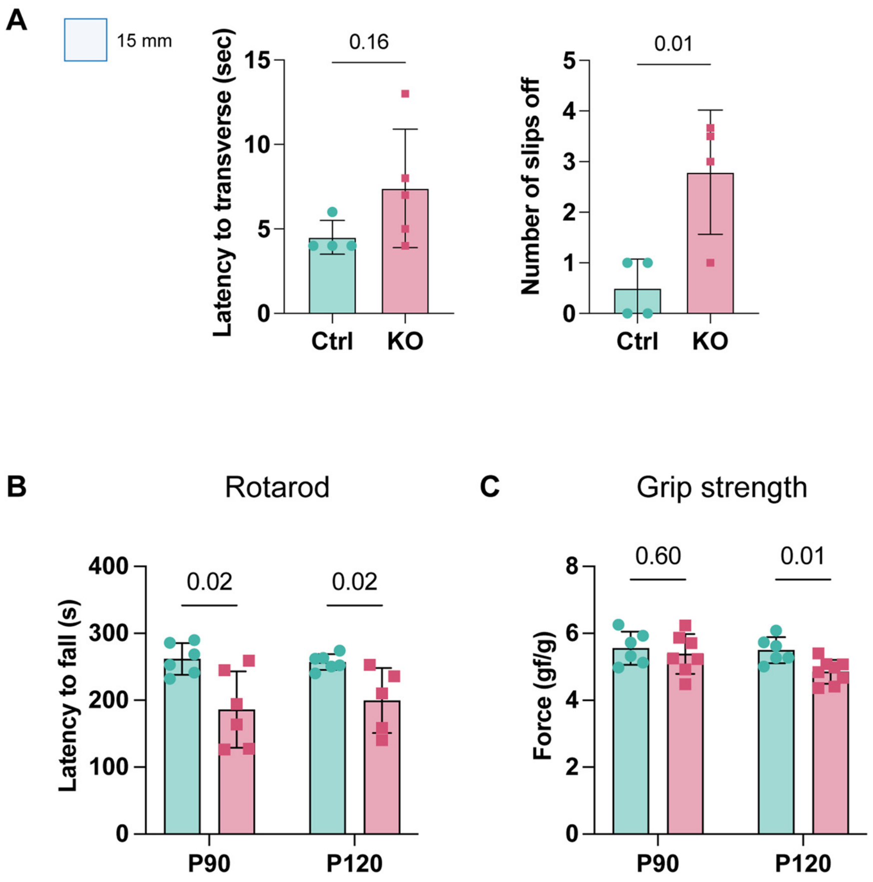

Figure EV4. Analysis of motor behavior in age-matched Ctrl and *Sertm2* mutant mice.

(Related to Fig. 5). (**A**) Time to transverse the beam (left) and the number of foot slips (right) on a 15-mm beam for Ctrl and *Sertm2* mutant (KO) mice. Data represent mean ± SD from $n = 4$–5 independent biological samples; unpaired two-tailed t test. $P = 0.16$ (Latency to transverse) and $P = 0.01$ (number of slips off) for Ctrl vs. KO. (**B**) Postnatal day (P) 90 and P120 Ctrl and KO mice were subjected to an accelerating rotarod test to measure motor performance. Latency to fall is shown. Data represent mean ± SD from $n = 5$–6 independent biological samples; unpaired two-tailed t test. P values for P90 and P120 (Ctrl vs. KO) were 0.02 and 0.02. (**C**) Motor strength was assayed according to grip strength of P90 and P120 Ctrl and KO mice. Grip strength measured in grams is displayed. Data represent mean ± SD from $n = 6$–7 independent biological samples; unpaired two-tailed t test. P values for P90 and P120 (Ctrl vs. KO) were 0.60 and 0.01.

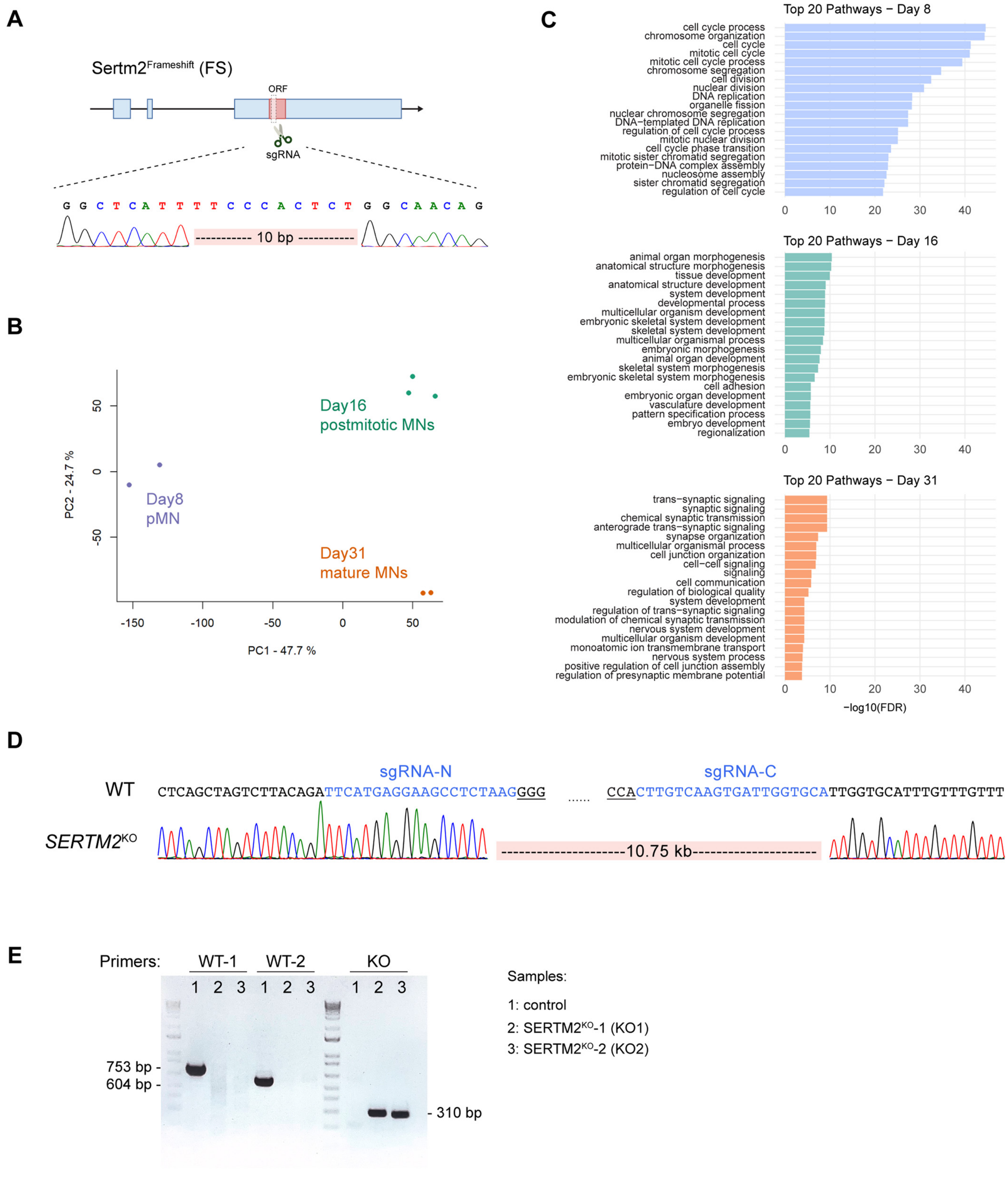
A
Sertm2Frameshift (FS)
ORF
sgRNA
G G C T C A T T T T C C C A C T C T G G C A A C A G
10 bp
B
Day16
postmitotic MNs
Day8
pMN
Day31
mature MNs
PC2 - 24.7 %
PC1 - 47.7 %
C
Top 20 Pathways − Day 8
cell cycle process
chromosome organization
cell cycle
mitotic cell cycle
mitotic cell cycle process
chromosome segregation
cell division
nuclear division
DNA replication
organelle fission
nuclear chromosome segregation
DNA−templated DNA replication
regulation of cell cycle process
mitotic nuclear division
cell cycle phase transition
mitotic sister chromatid segregation
protein−DNA complex assembly
nucleosome assembly
sister chromatid segregation
regulation of cell cycle
Top 20 Pathways − Day 16
animal organ morphogenesis
anatomical structure morphogenesis
tissue development
anatomical structure development
system development
developmental process
multicellular organism development
embryonic skeletal system development
skeletal system development
multicellular organismal process
embryonic morphogenesis
animal organ development
skeletal system morphogenesis
embryonic skeletal system morphogenesis
cell adhesion
embryonic organ development
vasculature development
pattern specification process
embryo development
regionalization
Top 20 Pathways − Day 31
trans−synaptic signaling
synaptic signaling
chemical synaptic transmission
anterograde trans−synaptic signaling
synapse organization
multicellular organismal process
cell junction organization
cell−cell signaling
signaling
cell communication
regulation of biological quality
system development
regulation of trans−synaptic signaling
modulation of chemical synaptic transmission
nervous system development
multicellular organism development
monoatomic ion transmembrane transport
nervous system process
positive regulation of cell junction assembly
regulation of presynaptic membrane potential
−log10(FDR)
D
sgRNA-N
sgRNA-C
WT
CTCAGCTAGTCTTACAGATTCATGAGGAAGCCTCTAAGGGG ...... CCACTTGTCAAGTGATTGGTGCATTGGTGCATTTGTTTGTTT
SERTM2KO
10.75 kb
E
Primers:
WT-1
WT-2
KO
1 2 3 1 2 3 1 2 3
753 bp
604 bp
310 bp
Samples:
1: control
2: SERTM2KO-1 (KO1)
3: SERTM2KO-2 (KO2)

 © The Author(s)

Figure EV5. Verification of Sertm2-FS ESC lines and characterization of SERTM2 in a human context, and verification of *SERTM2* knockout in a human iPSC line.

(Related to Figs. 6, 7). (**A**) Sanger sequencing confirmed the frameshift mutation in mouse Sertm2-FS ESCs. (**B**) The PCA plot of RNA-seq data from human ESC-derived MNs (MNX1::GFP). Samples represent specific time points during MN differentiation: Day 8 ($n = 2$), Day 16 ($n = 3$), and Day 31 ($n = 2$). (**C**) GO analysis was performed for the differentially expressed genes (DEGs) associated across stages of MN differentiation. The criteria for identifying DEGs are as follows: log2 fold change ≥ 1 and a false-discovery rate (FDR) < 0.05. (**D**) Sanger sequencing of the CRISPR/Cas9-mediated human *SERTM2* knockout human iPSC line (MNX1-tdT). The design of sgRNAs is highlighted in blue. (**E**) Genotyping of the human *SERTM2* KO line was performed using three distinct primer sets, as illustrated in Fig. 7F.
